# Optimizing the ingredients for imagery-based interpretation bias modification for depressed mood: Is self-generation more effective than imagination alone?^☆^

**DOI:** 10.1016/j.jad.2013.09.013

**Published:** 2014-01

**Authors:** Heike Rohrbacher, Simon E. Blackwell, Emily A. Holmes, Andrea Reinecke

**Affiliations:** a Department of Cognitive Psychology, Technische Universität Dresden, Dresden, Germany; b MRC Cognition and Brain Sciences Unit, Cambridge, UK; c Department of Psychiatry, University of Oxford, Oxford, UK

**Keywords:** Depression, Interpretation bias, Cognitive bias modification, Cognitive processing, Mental imagery

## Abstract

Negative interpretation is thought to be crucial in the development and maintenance of depression. Recently developed cognitive bias modification paradigms, intending to change these biases towards a more optimistic interpretation tendency (CBM-I), seem to offer new promising implications for cognitive therapy innovation. This study aimed to increase our knowledge of the underlying mechanisms of action of imagery-based CBM-I in the context of depressed mood. We therefore compared the efficacy of CBM-I requiring participants to imagine standardized positive resolutions to a novel, more active training version that required participants to generate the positive interpretations themselves. Fifty-four participants were randomly allocated to (1) standardized CBM-I, (2) self-generation CBM-I or (3) a control group. Outcome measures included self-report mood measures and a depression-related interpretation bias measure. Both positive training variants significantly increased the tendency to interpret fresh ambiguous material in an optimistic manner. However, only the standardized imagery CBM-I paradigm positively influenced mood.

## 1 Introduction

In day-to-day life, we encounter numerous examples of information that can be interpreted in more than one way, for instance facial expressions, feedback from others or physiological cues. How we disambiguate or make sense of these stimuli is important for how we further respond to the world. To interpret one's dialogue partner's yawning as a sign of boredom can obviously result in a different, even opposite emotion than attributing it to a simple lack of oxygen in the room. There is empirical consensus that negatively biased interpretation, defined as the tendency to consistently interpret ambiguous stimuli in a negative manner, is associated with dysphoria and depression and might play a key role in the development of disorders (e.g. Butler and Mathews, 1983; Eley et al., 2008; Hertel and El-Messidi, 2006; Lawson et al., 2002; Mathews and Mackintosh, 2000; Mogg et al., 2006; Reinecke et al., Submitted for publication; Rude et al., 2002, Wisco and Nolen-Hoeksema, 2010).

Cognitive bias modification paradigms (CBM; Grey and Mathews, 2000; Mathews and Mackintosh, 2000; Mathews and MacLeod, 2002) are computerized training procedures that encourage individuals to adopt a more positive (or negative) information processing style. For instance, in interpretation bias training procedures a more positive interpretation style can be trained by repeatedly presenting virtual scenarios that combine initially ambiguous information with a clearly positive outcome. An example by Holmes et al. (2006) is as follows: “You have started an evening class which is tough going. You are determined to succeed, and after a while, it becomes much easier and more enjoyable” (positive resolution in italics). Such procedures aim to identify the role of cognitive biases in the development of emotional disorders and have the potential to induce reductions in symptom severity by changing bias towards a more positive direction.

Although CBM-I has initially been developed for the training of anxiety-related bias, positive benefits of interpretation modification training could also be reported in the context of dysphoria and depression by a number of studies (Blackwell and Holmes, 2010; Holmes et al., 2008, 2009, 2006; Lang et al., 2012; Lothmann et al., 2011; Reinecke et al., Submitted for publication; Williams et al., in press). Positive bias modification has not only been shown to be effective in modifying the interpretation of fresh ambiguous information (e.g. Reinecke et al., Submitted for publication) in the trained direction, but it also ameliorates depressed mood and positively influences resilience to negative mood induction (e.g. Holmes et al., 2009).

A line of research (e.g. Holmes et al., 2009) has been interested in the most effective ingredients of these procedures. In line with prior research suggesting a special relationship between imagery and emotion (see Holmes and Mathews, 2005), Holmes et al. (2009) found imagery, rather than verbal processing to be crucial. Participants in the study were presented with 100 auditory scenarios that were initially ambiguous but consistently resolved in a positive manner. They were instructed to either imagine the events or listen to them while thinking about their meaning. Verbal processing of positive training material was not only less effective than imagery, but even led to paradoxical, negative mood responses. In other words, vividly imagining positive virtual events can improve your mood as well as how you interpret events, whereas only verbally thinking about the same positive contents can make you feel worse and negatively influence how you resolve ambiguities. Holmes et al. (2006, 2009) assumed that this surprising discrepancy could have been caused by different underlying mechanisms. While imagery might directly provoke emotion like a positive “as-if” experience, verbal processing might be perceived as less believable. It might more readily provoke a comparison with one's actual status quo, that could – especially in depressive individuals – turn out to be disadvantageous and thus mood deteriorating. However, the mental visualization of pleasant events alone does not guarantee positive emotion but depends on the precise task instruction. Holmes et al. (2008) demonstrated that only imagery from a field perspective (“through your own eyes”) improves affect as opposed to imagery from an observer perspective (“looking at you”), that could – like verbal processing – lead to adverse effects. Nelis et al. (2012) replicated the impact of imagery vs. verbal processing for positive CBM-I, but not of field vs. observer perspective, suggesting that this aspect requires further exploration. Based on these findings, effective interpretation bias modification can be obtained through a guided imagery training that provides participants with acoustically or visually presented descriptions of ambiguous virtual situations that are consistently combined with positive resolutions that need to be imagined from a field perspective (Holmes et al., 2008; Pictet et al., 2011).

Research in the context of anxiety-related interpretation bias has found active selection of meaning during the training to be critical for modifying subsequent emotional responses to new ambiguous stimuli (Hoppitt et al., 2010). In this study, participants were presented with threat-related ambiguous sentences that were negatively resolved by the final word, e.g. “You have decided to go caving even though you feel nervous about being in such an enclosed space. You get to the caves before anyone else arrived. Going deep inside the first cave you realize you have completely lost your *way*.” (negative resolution in italics). While participants in the passive group were presented with the entire passage, individuals in the active group were presented with only a fragment (one or more letters missing) of the final word (“….completely lost your *w--*.”) and therefore had to actively resolve the meaning by themselves (only one possible completion). Active selection of meaning was shown to be superior in modifying later emotional responses in a training-congruent way to images of new emotionally ambiguous descriptions presented after training than mere passive exposure. Hoppitt et al. (2010) suggested that this differential effect may be due to the induction of an implicit production rule, in which participants in the active condition continue to actively generate training-congruent meanings of subsequent ambiguous scenarios.

The self-perception theory (Bem, 1972) suggests that people infer (and modify) their attitudes, cognitions and emotions by observing their overt behaviours. According to this theory a person becomes, for instance, more committed to a certain attitude or general cognition if they have to argue on behalf of it (even when this position contradicts a previous attitude), in other words “as I hear myself talk, I learn what I believe” (Bem, 1972; Laird and Bresler, 1992; Miller and Rollnick, 2002). This principle has been supported by numerous studies. For example, manipulated facial expressions can trigger changes in emotion (e.g. Laird and Bresler, 1992) as well as changes in attitudes (racial bias) as assessed by the Implicit Association Test (Ito et al., 2006). Sharot et al. (2010) studied participants who rated different vacation destinations both before and after making a blind choice that could not be guided by pre-existing preferences. Their results demonstrated that choices not only reveal preferences, but also shape them even when decisions were made randomly. Interestingly, change in preferences was observed only when participants believed they had been instrumental in making a decision, and not when the decision was made by a computer. We hypothesised that the self-perception principle could also be relevant for the modification of cognitive interpretation bias and be integrated in CBM-I procedures and potentially enhance its effect by instructing participants to not only imagine but to positively complete the initially ambiguous scenarios by themselves.

The aim of this study was to develop a new, more active variant of CBM-I and test its impact on positive mood and interpretation bias in comparison to a control group as well as imagery CBM-I. The purpose of this comparison was to further our knowledge about the mechanisms of action underlying successful CBM-I and to optimize its ingredients to enhance its future therapeutic potential. The positive imagery CBM-I can be described as a standardized guided imagery training that provides participants with auditorily presented descriptions of ambiguous virtual everyday life situations that are consistently combined with positive resolutions (e.g., Holmes et al., 2006). The new training variant, however, instructs participants to not only imagine but to positively complete the initially ambiguous scenarios themselves by speaking one or two phrases into a microphone. As participants in the new training variant have to invent positive resolutions themselves, we expected that they would perceive the scenarios as more authentic than participants in the standardized guided imagery training. Further, the self-generation of positive resolutions (and its subsequent vocalisation) can be seen as a more active process than guided imagery and in terms of the self-perception theory be regarded as “overt behaviour” that could lead to a (greater) modification of cognitions (interpretation bias) and internal states (mood). We therefore hypothesised that the self-generation variant would be more effective in changing interpretation bias and mood towards a more positive direction, based on the principles of self-perception as well as prior findings by Hoppitt et al. (2010).

## 2 Method

### 2.1 Participants

54 participants (13 male, 41 female) took part in the study (age in years: *M*=22.0, *SD*=2.9). They were recruited via online advertisements at the website of the Technische Universität Dresden. The majority of them were undergraduate students. One third of the participants (29.6%) in this study showed BDI-scores above the clinical cut-off score with 22.2% reporting symptoms of mild depression (BDI-scores: 14–19) and 7.5% reporting symptoms of moderate depression (BDI-scores: 20–28). Reimbursement for participation consisted of either a small participation fee (5€) or course credits.

### 2.2 Materials

#### 2.2.1 Questionnaire measures

The German version of the Beck Depression Inventory II (BDI-II; Beck et al., 1996; Hautzinger et al., 2006) was used to measure current levels of depressive symptoms. The trait version of the German version of the Spielberger State-Trait Anxiety Inventory (STAI-T; Laux et al., 1981; Spielberger et al., 1983) was given to assess trait anxiety. State positive and negative affect were measured using the German version of the Positive and Negative Affect Schedule (PANAS; Krohne et al., 1996; Watson et al., 1988). The 20-item scale was administered with the short-term time instruction (“indicate to what extent you feel this way now”) and rated from 1 (not at all) to 5 (extremely).

#### 2.2.2 Interpretation bias measure

Interpretation bias was measured using the Ambiguous Scenarios Test for depression-related interpretation bias (AST-D-II; Rohrbacher et al., Submitted for publication). This was developed from the Ambiguous Scenarios Test (AST; Berna et al., 2011; Holmes et al., 2009; Mathews and Mackintosh, 2000), where ambiguous situational descriptions are presented that allow either a positive or a negative outcome interpretation (e.g. It's the morning of your birthday. The postman comes down the street with their bag). The outcome measure is the emotional valence rating of each description made by participants on a 9-point scale, ranging from extremely unpleasant to extremely pleasant. The AST-D-II consists of two 15-item parallel questionnaires in German, one of which was presented before and after CBM-I, respectively, with the order being pseudo-randomised and counterbalanced between groups. Both versions have been shown to possess high psychometric properties, including high-parallel-reliability (Rohrbacher et al., Submitted for publication).

#### 2.2.3 Interpretation bias modification training

Participants were randomly assigned to (1) standardized CBM-I-, (2) self-generation CBM-I, or (3) a control group (CG). Using the CBM-I paradigm described by Holmes et al. (2006), participants in all three groups were presented with 50 auditory scenarios. A definition of mental imagery was given. Then participants were instructed to vividly imagine these scenarios from a field-perspective, and to try not to engage in verbal thoughts about the scenarios, such as making comparisons between the scenario and themselves (for details on instructions with practice examples, see Holmes et al. (2008, 2009)). Across the three training conditions, the first part of a specific scenario was identical (e.g. *“*You are on your way to the photographer to pick up your new portrait pictures. As you look at the photos you are quite surprised.”). This part was also ambiguous in that it allowed either a positive or a negative outcome. Each statement lasted about 5–10 s. Scenarios in all groups were recorded using the same female voice and presented stereo-phonically through headphones.

The second part of the scenarios differed between training conditions. In the standardized CBM-I condition, the auditory description continued directly on with the positive resolution (e.g. “ The pictures are much better than you expected.”). Participants had to vividly imagine the complete scenario including its positive resolution (no time limit was given) and then had to press a “continue”—button to start the next scenario. Participants in the condition self-generation CBM-I were presented the initial part of the scenario. They were instructed (before training) to vividly imagine the auditorily presented scenario and to resolve its ambiguity by imagining a positive completion by themselves. After the imagery part (no time limit was given) participants had to press a “start”-button and were asked to describe their actively generated positive resolution by speaking a few words into a microphone (all completions were recorded in order to check for compliance). Individuals had to press a “continue”—button to start the next scenario. Participants in the control group were merely presented the first part of the scenario with no resolution. They had to vividly imagine each scenario and had to press a “continue”—button to start the next scenario.

#### 2.2.4 Filler

Following previous studies (e.g. Holmes et al., 2006), in order to equalize mood levels after training between the three groups (before the application of the interpretation bias test), participants were presented with a series of classical music extracts for a 10-min interval after the interpretation bias training. Participants were asked to rate the extracts with respect to their pleasantness.

#### 2.2.5 Manipulation checks

During the training, participants gave ratings at the end of each 10th scenario regarding the previously presented scenario. Participants were asked to rate (a) to what degree they had been able to vividly imagine the scenario (vividness) (b) as how realistic they had experienced the presented scenario (authenticity) and (c) as how pleasant they had perceived the presented scenario (pleasantness). Responses were rated on a 9-point scale, ranging from 1 (not at all) to 9 (extremely).

#### 2.2.6 Memory questions/expectancy beliefs

Furthermore, participants were given five memory questions at the end of the session (e.g. “What present did you buy for your partner (in one of the presented scenarios)?”). These questions were part of the cover story and additionally served as a filter to exclude participants with insufficient ability to concentrate on the training. Since imagery has been associated with enhanced learning and memory effects (e.g. Schwartz and Heiser, 2005), we expected participants to recollect the majority of the memory questions. As previous pilot-testing revealed that most pilot-participants were able to correctly answer 4 to 5 of the memory questions, an exclusion cut-off of less than three correct answers was determined a priori. None of the participants fell below that cut-off. In addition, participants were asked to guess the purpose of the study (“In your opinion, what is the purpose of this study?”) after completion of the experiment in order to estimate possible demand or expectancy effects.

### 2.3 Procedure

In order to decrease expectancy as well as demand effects, participants were provided with a cover story. They were told that the purpose of the study was to examine the association between memory effects and spatial representations, and that they would have to answer several questions concerning their mental images after an imagery-task (*experimental phase*). After having given informed consent and being randomly assigned to one of the three training-conditions, participants completed BDI-II, STAI-T, PANAS, as well as the pre-test of the AST-D-II. After the *experimental phase (CBM-I)*, they again completed the PANAS. This state questionnaire was given for a third time after the 10 min *filler task*. Participants then completed the post-test AST-D-II. They completed the *manipulation check ratings* (vividness, authenticity, pleasantness) during the training. At the end of the study, they answered five *memory questions* and were asked about their *expectancy beliefs* regarding the aim of the study.

## 3 Results

### 3.1 Comparison of participants in the three training conditions at baseline

Questionnaire scores of all baseline measures are depicted in Table 1. The three CBM groups were not different in terms of gender ratio, *X*^2^(2)=0.20, *p*=.90, mean age (*F*(2,53)=0.20, *p*=.82), depressive symptoms (BDI-II; *F*(2,53)=2.20, *p*=.12), trait anxiety (STAI-T; (*F*(2,53)=1.30, *p*=.28), mood (PANAS-positive; *F*(2,53)=0.45, *p*=.64; PANAS-negative; *F*(2,53)=0.94, *p*=.40), nor interpretation tendencies (pre-training AST-D-II; *F*(2,53)=0.40, *p*=.67).

### 3.2 Effects over training—Changes in mood measured with the PANAS

**Fig. 1:**
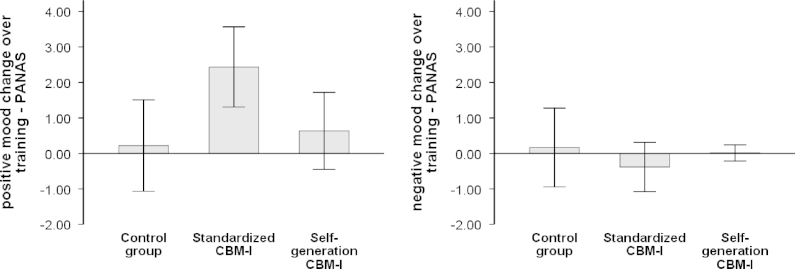
Mood change scores are calculated as the difference between the PANAS post-training score and the PANAS pre-training score; positive scores reflect an increase on the measure, negative scores a decrease. Error bars show the standard deviation of the mean. *Note:* PANAS=positive and negative affect schedule.

Two 2 x 3 repeated measures ANOVAs with the within-factor time (pre- vs. post training) and the between-factor group (CG, standardized CBM-I, self-generation CBM-I) were calculated using the pre- and post-test scores of the PANAS as the dependent variables, separated for the positive and the negative PANAS subscale (Table 1, Fig. 1). *Negative subscale:* There was no significant main effect of the factors time, *F*(1,51)=0.09, *p*=.76, or group, *F*(2,51)=1.15, *p*=.32, nor a significant interaction, all *F*(2,51)=0.54, *p*=.58, indicating that none of the trainings lead to a change in negative mood. *Positive subscale:* There was no main effect of group, *F*(2,51)=1.27, *p*=.29, but a significant effect of time, *F*(1,51)=10.56, *p*<.01, *η*^*2*^_*p*_=.17, as well as a significant interaction between time and group, *F*(2,51)=4.05, *p*=.02, *η*^*2*^_*p*_=.14, indicating differential changes in positive mood in the three groups during training. Additional paired-samples *t*-tests comparing pre- and post-CBM mood scores, separated for the three CBM groups, indicated a significant increase of positive mood during standardized CBM-I, *t*(17)=4.32, *p*<.01, whereas no change was found in self-generation CBM-I, *t*(17)=1.17, *p*=.26, and the control group, *t*(17)=0.35, *p*=.73. There was a trend level difference between the three groups' levels of positive affect post-CBM, *F*(2,51)=2.43, *p*=.09. In summary, our analysis revealed no changes in negative mood in any of the training conditions, but an increase of positive mood in the standardized CBM-I used in earlier studies.

#### 3.2.1 Mood change during the filler task

To assess whether the mood improvement during training in the standardized CBM-I group survives a neutral filler task or is rather transient, we ran an additional 2×3 repeated measures ANOVA with the within-factor time (pre- vs. post filler) and the between-factor group, using the pre- and post-filler task scores of the positive subscale of the PANAS as the dependent variable. The was no significant main effect of group, *F*(2,51)=2.02, *p*=.14, but a significant effect of time, *F*(1,51)=57.36, *p*<.01, *η*^*2*^_*p*_=.53, and a significant interaction between time and group, *F*(2,51)=12.16, *p*<.01, *η*^*2*^_*p*_=.32, indicating differential changes in mood in the three groups during the filler task. Follow-up paired-samples *t*-tests, comparing pre- and post-filler positive mood, indicated a significant increase of positive mood in the control group, *t*(17)=9.22, *p*<.01 and self-generation CBM-I, *t*(17)=3.69, *p*<.01, whereas no change was found in standardized CBM-I, *t*(17)=1.00, *p*=.33. In summary, our analysis revealed no mood transition in standardized CBM-I, but mood changes in self-generation CBM-I as well as in the control group.

### 3.3 Mood after the filler task

Two one-way ANOVAs, separated for the negative and the positive subscale of the PANAS, were run for the scores recorded after the filler task to compare mood across the three CBM groups. No significant differences between the three training groups were found, neither for negative nor positive affect scores, both *F*(2,53)<1.65, both *p*>.20. This indicates that the groups were comparable prior to the administration of the bias tasks.

### 3.4 Effects over training—Interpretation bias measured via AST-D-II

**Fig. 2:**
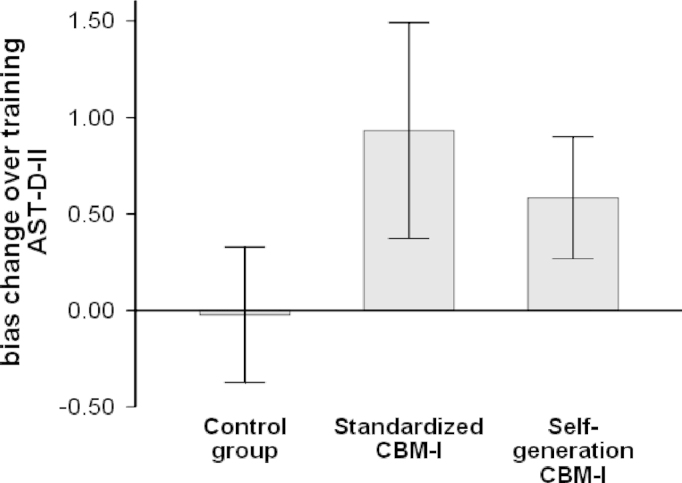
Bias change scores are calculated as the difference between the AST-D-II post-training score and the AST-D-II pre-training score; positive scores reflect an increase in a positive direction. Error bars show the standard deviation of the mean. *Note:* AST-D-II=Ambiguous scenario test II.

A 2×3 repeated measures ANOVA with the within-factor time (pre- vs. post training) and the between factor group (CG, standardized CBM-I, self-generation CBM-I) with the pre- and post-training scores of the AST-D-II as the dependent variables was run (Table 1, Fig. 2). The analysis yielded no main effect of group, *F*(2,51)=0.30, *p*=.74, but a significant effect of time, *F*(1,51)=16.68, *p*<.01, *η*^*2*^_*p*_=.25, and a significant interaction between time and group, *F*(2,51)=5.23, *p*<.01, *η*^*2*^_*p*_=.17, indicating differential changes in interpretation bias in the three groups during training. Additional paired samples *t*-tests separately conducted for each of the three groups indicated a significant increase of positive interpretation bias during training in the standardized CBM-I group, *t*(17)=3.69, *p*<.01, and the self-generation CBM-I group, *t*(17)=3.33, *p*<.01, whereas no change was found in the CG, *t*(17)=0.13, *p*=.90. Independent-samples *t*-tests suggested no difference between the training effect scores of standardized CBM-I and self-generation CBM-I, *t*(34)=1.08, *p*=.29, indicating these two trainings as equally effective in changing interpretation style towards a more positive direction. There was no significant difference in AST-D-II scores post-training between the three groups, *F*(2,51)=1.98, *p*=.15. In summary, participants in the positive training conditions, but not in the control group, showed a similar increase of positive interpretation as measured with the AST-D-II.

### 3.5 Manipulation checks/memory questions/expectancy beliefs

Using one-way ANOVAs, the groups did not differ regarding their ratings of the vividness and the authenticity of the situation samples, nor their imagery-related memory performance, all *F*(2,53)<1.82, *p*>.17. However, a significant difference was found regarding their pleasantness ratings, *F*(2,53)=1.82, *p*<.01, *η*^*2*^_*p*_=.54. Subsequent post-hoc pair wise comparisons (Bonferroni) revealed that the control group rated the scenarios as significantly less pleasant than did the two positive training conditions (both *p*<.01). None of the participants correctly guessed the purpose of the study when asked at the end.

## 4 Discussion

The goal of this study was to design and evaluate a new self-generation variant of imagery CBM-I and test its effectiveness in comparison to a control group as well as standard or guided imagery CBM-I. The purpose of this comparison was to increase the knowledge about the mechanisms of action behind this cognitive bias modification procedure. Our self-generation version of CBM-I increased positive interpretation, whereas no such change was seen in the control condition. We had assumed that self-generation CBM-I would be more effective than the standardized variant, based on the principles of self-perception as well as prior findings by Hoppitt et al. (2010), but in fact our findings did not support this prediction. Contrary to hypothesis, the effect of self-generation CBM-I on interpretation bias was not superior compared to the standardized CBM-I. Moreover, the new CBM-I variant had no effect on either positive or negative mood. In contrast, the type of instruction used in earlier studies led to a significant increase in positive mood. These results do not confirm the expected superiority of a more active CBM-I version involving self-generation of positive resolutions in enhancing mood and interpretation, but rather advocate the use of standardized scripts for imagery-based CBM-I to target depressive mood.

How can these findings be explained? We had suggested that the self-generation of positive resolutions (and its subsequent vocalisation) as a more active and overt behaviour would lead – according to the self-perception theory – to greater changes in interpretation bias as well as mood. We had expected that observing themselves generating the (positive) resolutions for the scenarios would facilitate participants internalising these resolutions as their own expectations for the outcomes of the imagined situations. From a self-perception theory perspective, the lack of additional effect on modification of interpretation bias of this self-generation could perhaps be explained by the idea that mental imagery from a field perspective is like an “as-if” experience (cf. Holmes et al., 2009). That is, even if the imagery is guided, when participants imagine themselves engaging in and experiencing the positive outcomes, they modify their cognitions in line with what they experience themselves thinking, feeling, and doing—albeit in their minds' eye. It may be that the process of creating a scenario resolution to imagine and vocalising it verbally adds nothing on top of this. Consistent with this, analysis of ratings during the training revealed that participants in self-generation CBM-I did not perceive the training situations as more authentic than those in standardized CBM-I. That is, while we had expected that inventing the positive resolutions for themselves might lead to participants experiencing the scenarios as more authentic (i.e. realistic) this was not in fact the case. We suggest that this finding might be because imagery in field perspective – whether guided or self-generated – precludes the kind of evaluative thought processes that might lead to finding the scenarios unrealistic (cf. Holmes et al., 2009). Therefore, attempting to increase the believability by other means (e.g. self-generation of the resolution) may not add anything above encouraging vivid field-perspective imagery.

Interestingly, the finding of similar effects on interpretation after self-generated and standardized CBM-I could be seen as also contrasting with previous research, showing stronger effects of “active” compared to “passive” procedures in modifying later responses to new emotionally ambiguous descriptions (Hoppitt et al., 2010). However, importantly, substantial differences in study designs might account for this apparent contradiction. First, the present study explored the potential of two depression-specific positive CBM-I versions in altering interpretation, while the previous study investigated anxiety-specific negative CBM-I. Furthermore, while in our more “active” training variant (self-generated resolutions) participants had to freely invent positive resolutions to then incorporate these into mental imagery, Hoppitt et al. (2011) asked their participants to resolve a fragmented word at the end of a written scenario, with only one solution being possible. Finally, in contrast to the “passive” condition used by Hoppitt et al. (2011), the CBM-I using standardized scripts does require participants to actively engage in the emotional valence of the training material, by generating a field-perspective mental image of the situation described. Such differences in target disorder, CBM-I complexity and demand, and the use of mental imagery might be relevant for the interpretation of results.

Why was there an unexpected lack of mood improvement during our self-generation CBM-I training as opposed to the significant increase of positive mood in standardized CBM-I, that even remained stable over a filler task? Perhaps the generation of positive outcomes may (at least in this study) place a higher demand on concentration and performance, reducing any potential increase in positive affect. The requirement to concentrate on self-generating an ending may have also disrupted participants' absorption in the positive imagery, reducing its emotional impact, or resulted in more evaluative post-processing of the image via the requirement to monitor the valence of the ending generated. That is, having to “think about” the positive endings may have distracted from the “as if” processing that characterizes mental imagery, and promoted more comparative, evaluative processing (cf. Holmes et al., 2009). The two training groups did not differ regarding their pleasantness-ratings, we therefore assume that the self-generated resolutions were perceived as equally positive as the standardized resolutions. However, the pleasantness-ratings do not allow conclusions about the objective positivity. This could be an interesting aim for a follow-up study in which the self-generated resolutions are used as guided solutions in the standardized condition to control for objective positivity. It is also worth noting that the explicit information in the self-generation condition that participants would have to generate positive resolutions to all of the training scenarios may have reduced the initial ambiguity of the stems, as participants would be aware from the start of each scenario that it would end positively (cf. Clarke et al., in press).

What might be the significance of the greater increase in positive mood seen in the standardized imagery CBM-I? Although the main aim of the CBM-I paradigm may be to train more positive interpretation, if the training sessions also boost positive mood then this may have significant implications for future clinical application in depression. Positive affect may be an important determinant of clinical outcome in depression, with lack of positive affect predicting poorer prognosis (e.g. Morris et al., 2009), and early improvement in positive affect predicting response to antidepressant treatment (Geschwind et al., 2011). Furthermore, there is some preliminary evidence that repeated practice in upregulating the neural areas involved in generating positive emotions may provide a promising route for novel treatment development (Linden et al., 2012). Thus, although the paradigm's primary aim may not be to boost positive affect, this aspect of it may help increase its potential clinical impact. On a very practical level, if a depressed individual was completing the CBM-I in their home environment, then even a transient increase in positive mood following completion of a training session could potentially have a positive impact on subsequent activities. Increasing our understanding of the regulation of positive affect in depression may help to capitalise on such transient increases and increase possible therapeutic benefits (Raes et al., 2012; Werner-Seidler et al., 2013). Finally, even if the increase in positive affect had no significant clinical impact, the expectation of a boost in positive mood may enhance motivation to engage with a CBM-I treatment schedule. Our finding that the mood improvement in standardized CBM-I was maintained over a filler task seems very encouraging in terms of stability. However, follow-up studies are needed to clarify prolonged mood effects or their sustained effect after a negative mood induction to verify clinical utility.

On the basis of prior research (e.g. Holmes et al., 2006, 2009) and supported by our findings we conclude that imagery CBM-I using standardized scripts, as in previous studies, is the most useful form to pursue. This is perhaps a promising outcome for clinical translation, as people with depressed mood may find it particularly difficult to generate positive interpretations (cf. Wisco and Nolen-Hoeksema, 2010), and thus a standardized imagery CBM-I paradigm may be easier to engage in successfully. Imagery techniques may work relatively well at an emotional level (Holmes and Mathews, 2005), as opposed to cognitive therapy that focuses on rational and verbal techniques such as identifying distorted thinking and challenging dysfunctional beliefs. Further, imagery has been associated with enhanced learning and memory effects (e.g. Schwartz and Heiser, 2005) and may have potential longer term effects. It may be that efforts to increase the effectiveness of imagery-based CBM-I should focus on enhancing the use of imagery in the training, or on refining the composition of the standardized training scenarios, rather than by incorporating non-imagery processing requirements into the tasks.

A few limitations of the study need to be highlighted. First, our assessments relied on self-report, involving the risk of response bias. However, as none of the individuals in our sample correctly guessed the study purpose, the influence of demand or expectancy effects on results is rather unlikely. Furthermore, preliminary studies using more involuntary, psycho-physiological measures of interpretation provide further evidence for the association between interpretation bias and depression (e.g. Moser et al., 2012). Nevertheless, future studies into the effect of CBM on mood and interpretation bias are encouraged to incorporate more objective measures. Second, although participants rated vividness of imagery equally in all three conditions, we did not measure the participants' baseline imagery ability and so do not know whether these ratings were equally accurate in all conditions. Follow-up studies should therefore measure trait imagery and also ask participants to provide further ratings of their imagery (e.g. “positiveness”) in order to obtain a fuller picture of their experience of it. A third issue that needs to be considered refers to the generalizability of the present results to patients with depressive disorders and to the interpretation of real-life situations. Given the fact that one third of the participants (29.6%) in this study showed BDI-scores above the clinical cut-off score (BDI-scores>14) makes transfer to clinical samples very promising. Future studies should investigate the longer term impact of the immediate effects of the CBM-I on bias and mood, and their importance in both near and far transfer of the training (Hertel and Mathews, 2011).

In summary, the current study adds further support to the potential therapeutic significance of guided CBM-I-based imagery techniques and evaluated a new, self-generation variant of CBM-I.

## Role of funding source

Although some of the authors are supported by funding from sources Elsevier has an agreement with, this research has been supported by funds other than these.

## Conflict of interest

None of the authors have any conflict of interest to report.

## Figures and Tables

**Table 1:** Characteristics of participants and effects over training.

	Control group (*n*=18)		Standardized CBM-I (*n*=18)		Self-generation CBM-I (*n*=18)	
	M	SD	M	SD	M	SD
Age	22.2	2.7	21.6	3.5	22.0	2.6
% Female	78.0	0.4	72.0	0.5	78.0	0.4
STAI Trait	45.8	5.1	43.9	6.3	46.7	4.3
BDI-II	8.7	5.6	10.3	6.7	12.8	5.0

PANAS-positive						
Pre-training	31.94	5.88	32.77	4.25	31.14	5.17
Post-training	32.17	6.25	35.21	3.81	31.77	5.00
After filler	33.00	6.05	35.26	3.92	32.40	4.81
PANAS-negative						
Pre-training	12.89	3.45	11.73	1.99	12.44	1.97
Post-training	13.06	5.03	11.34	2.01	12.45	2.08
After filler	12.83	3.78	11.29	1.55	12.34	1.93

AST-D-II						
Pre-training	0.83	0.74	0.55	1.19	0.74	0.93
Post-training	0.81	0.87	1.48	1.32	1.33	0.93

Ratings during training						
Vividness	1.98	1.55	2.78	1.15	1.99	1.62
Authenticity	1.24	1.37	1.77	1.00	1.40	1.40
Pleasantness	−0.70	0.98	2.73	1.63	1.66	1.36
Memory questions after training (% of correct answers)	4.39	0.50	4.22	0.43	4.44	0.51

*Note:* STAI=State-trait anxiety inventory; BDI-II=Beck depression inventory II; PANAS=Positive and negative affect schedule; AST-D-II=Ambiguous scenario test II.
